# Joint Association of Dietary Pattern and Physical Activity Level with Cardiovascular Disease Risk Factors among Chinese Men: A Cross-Sectional Study

**DOI:** 10.1371/journal.pone.0066210

**Published:** 2013-06-19

**Authors:** Dong Wang, Yuna He, Yanping Li, Dechun Luan, Fengying Zhai, Xiaoguang Yang, Guansheng Ma

**Affiliations:** 1 National Institute for Nutrition and Food Safety, Chinese Center for Disease Control and Prevention, Beijing, China; 2 Department of Nutrition, Harvard School of Public Health, Boston, Massachusetts, United States of America; 3 Division of Human Nutrition, Wageningen University, Wageningen, the Netherlands; 4 Liaoning Provincial Center for Disease Control and Prevention, Shenyang, Liaoning Province, China; Charité University Medicine Berlin, Germany

## Abstract

The purpose of this cross-sectional study was to investigate the joint associations of physical activity level (PAL) and dietary patterns in relation to cardiovascular disease (CVD) risk factors among Chinese men. The study population consisted of 13 511 Chinese males aged 18–59 years from the 2002 China National Nutrition and Health Survey. Based on dietary data collected by a food frequency questionnaire, four dietary patterns were identified and labeled as “Green Water” (high consumption of rice, vegetables, seafood, pork, and poultry), “Yellow Earth” (high consumption of wheat flour products and starchy tubers), “New Affluent” (high consumption of animal sourced foods and soybean products), and “Western Adopter” (high consumption of animal sourced foods, cakes, and soft drinks). From the information collected by a 1-year physical activity questionnaire, PAL was calculated and classified into 4 categories: sedentary, low active, active, and very active. As compared with their counterparts from the New Affluent pattern, participants who followed the Green Water pattern had a lower likelihood of abdominal obesity (AO; 50.2%), hypertension (HT; 37.9%), hyperglycemia (HG; 41.5%), elevated triglyceride (ETG; 14.5%), low HDL (LHDL; 39.8%), and metabolic syndrome (MS; 51.9%). When compared to sedentary participants, the odds ratio of participants with very active PAL was 0.62 for AO, 0.85 for HT, 0.71 for HG, 0.76 for ETG, 0.74 for LHDL, and 0.58 for MS. Individuals who followed both very active PAL and the Green Water pattern had a lower likelihood of CVD risk factors (AO: 65.8%, HT: 39.1%, HG: 57.4%, ETG: 35.4%, LHDL: 56.1%, and MS: 75.0%), compared to their counterparts who followed both sedentary PAL and the New Affluent pattern. In addition, adherence to both healthy dietary pattern and very active PAL presented a remarkable potential for CVD risk factor prevention.

## Introduction

Cardiovascular disease (CVD) is the leading cause of death in the world [1]. In China, accompanying the rapid economic development and urbanization, the prevalence of CVD has increased dramatically from 3.1% in 1993 to 8.6% in 2008 [2]. To curb this trend, one of the most important [3] and cost-effective [4] strategies is to control CVD risk factors such as hypertension (HT), dyslipidemia, metabolic syndrome (MS), and obesity, consequently, preventing and delaying the development of subclinical atherosclerosis and other myocardial and vascular changes over time [3] and avoiding ultimate onset of CVD. Compared with costly pharmacologic treatments, lifestyle adjustment, such as adopting regular physical activity and healthy diet, may achieve better outcomes for the reduction of CVD risk and be more widely applicable to a broader population [3]. However, most previous studies only examined the relationship between CVD risk factors and crude estimates of diet (eg. a specific nutrient) or physical activity (eg. leisure time physical activity times per day), explaining only a portion of the morbidity of CVD risk factors and limiting a detailed exploration of the relationship between CVD risk factors and lifestyle factors [5], [6]. The research community now recognized that we must refine methods of measuring these two lifestyle behaviors [5], [6] because diet and physical activity are such complex exposure variables. As a result, dietary pattern and physical activity level (PAL) analysis, which provide overall estimation of these two lifestyle factors, have begun to emerge in epidemiology research [5]–[8].

Dietary pattern analysis assesses the total diet with consideration for the interactive or synergistic effects of nutrients and allows researchers to capture diet-disease relationships without knowing the specific food or nutrient involved [5]. Some observational studies and clinical trials among Western populations have suggested several healthful dietary patterns that appear to be effective at preventing CVD risk factors [5], [9]–[13]. Based on the data from the 2002 China National Nutrition and Health Survey (CNNHS), our previous study [14] identified the major dietary patterns in China, which were very different from the Western dietary patterns. To evaluate total amount of physical activity, previous studies have developed the PAL model [7], [15], which is a function of physical activity’s intensity, duration and frequency, and related PAL with CVD risk [16]–[18]. Also based on the 2002 CNNHS data, we developed the Chinese version of PAL and associated PAL with prevalence of obesity among the Chinese population [19]; however, to date, we do not know if and how the dietary patterns and PAL are related to CVD risk factors in a nationally representative sample of Chinese adults. Furthermore, in most of the previous studies mentioned above, dietary pattern or PAL was studied individually. However, some recent studies suggested that combined low-risk lifestyle habits were often correlated and more effective on the prevention of some chronic diseases [20]–[22]. In observational and interventional studies among western populations [20]–[23], when combined for evaluation, optimal dietary pattern and PAL appeared to reduce a great majority of CVD risk factor cases. However, the joint association of dietary pattern and PAL with CVD risk factors among Chinese populations is largely unknown. To address these questions, we investigated both the individual and joint associations of dietary pattern and PAL in relation to CVD risk factors in a nationally representative sample of Chinese male adults. It is quite possible that extremely low prevalence may result in insufficient statistical power and make it difficult to detect the associations between lifestyle and CVD risk factors. This phenomenon has already been observed among Chinese females by a previous study which examined the associations between lifestyle risk factors and chronic disease. For example, in a case-control study in China, due to the low prevalence of women’s smoking and alcohol drinking, Ji et al. stated the difficulty of obtaining sufficient statistical power to build significant association between lifestyle factors and colorectal cancer [24]. The current study only focused on male Chinese, because of previous evidence and existing findings of low prevalence of lifestyle risk factors in female Chinese [19], [25]

## Methods

### Study population

The 2002 CNNHS is a nationally representative cross-sectional study on nutrition and non-communicable chronic diseases, covering all 31 provinces, autonomous regions, and municipalities directly under the central government throughout China (except Taiwan, Hong Kong, and Macao). In this survey, a stratified, multistage probability cluster sampling design was used, as described in detail previously [26]. On the basis of socioeconomic characteristics, the whole country was divided into six categories. As described elsewhere [27], in the first stage of sampling, 22 counties were randomly selected from each of the six socioeconomic regions. In the second stage, three townships were randomly selected from each of the selected counties. From each of these townships, two residential villages were randomly sampled, and 90 households were then randomly sampled from each village for physical and medical examinations. Approximately one-third of all households were selected to participate in a dietary survey and blood draw. We informed and explained our study methods, the benefits and adverse reactions, the objectives of this study, and the definition of ethnicity to all subjects. Written consent was obtained from every subject before physical examination, questionnaire administration, and blood sample collection. In the current study, among all 73 260 male participants aged 18–59 years in 2002 CNNHS, 13 511 male participants aged 18–59 years were selected from the dietary assessments and blood sample collection. This study was approved by the Ethics Committee of the Chinese Center for Disease Control and Prevention.

### Dietary assessment

A validated, semi-quantitative food frequency questionnaire (FFQ) was used to investigate the dietary intake in the previous year before the study [28] and recorded both the frequency and the quantity of the 33 types of food categories.

The construction of dietary patterns using factor analysis combined with cluster analysis was described in detail elsewhere [14]. Briefly, we first applied principal component analysis to identify 4 groups of interrelated food categories. Then the factor scores were used in a cluster analysis and revealed a 4-cluster solution. The first cluster, the Yellow Earth pattern, represented a typical traditional diet in northern China which was characterized by high intake of wheat flour products and starchy tubers, combining with low consumption of protein products such as pork, beef, poultry, seafood, or milk and milk products. The second cluster, the Green Water pattern, represented a typical traditional diet in southern China, characterized by high intake of rice, vegetables, seafood, pork, and poultry. The third cluster, the New Affluence pattern, was characterized by living in urban areas, having a higher intake of animal sourced foods and soybean products. The fourth cluster, the Western Adaptor pattern, was characterized by a high consumption of animal sourced foods, cakes, and drinks. Food consumption and nutrient intake for participants with each dietary pattern were described in Tables S1 and S2.

### Physical activity level

Trained investigators collected information on physical activity using a 1-year physical activity questionnaire. Physical activity was categorized into five domains: occupational, leisure time, transportation, household work and sedentary activities (including watching television, using a computer, playing video games and reading during leisure time). Then the frequency and duration of each domain were recorded. According to the Compendium of Physical Activities, the intensity of each activity in the questionnaire was coded [15]. In the present study, we employed PAL, which describes the ratio of total energy expenditure (TEE) divided by basal energy expenditure (BEE) extrapolated to one day, to evaluate the participants’ total amount of physical activity. According to the recommendation from Institute of Medicine (IOM) [7], PAL was calculated by the following equations:



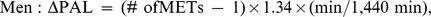
,



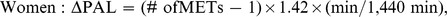
,
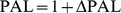



where METs is Metabolic Equivalents, which is defined as a rate of oxygen (O^2^) consumption of 3.5 ml/kg/min in adults.

In our study, reported physical activities performed over the course of 1 year were assigned a METs value (METs) based on the Compendium of Physical Activities [15]. According to IOM’s recommendation [7], PAL was classified into four categories: sedentary PAL (1.00–1.39); low active (PAL 1.40–1.59); active (PAL 1.60–1.89); and very active (PAL 1.90–2.50) [29]. We also explained the 4 PALs in the Text S1.

### General information and health behavior

A household general information questionnaire collected participants’ education level and annual family income. Health behavior risk factors included current smoking status and alcohol consumption. Participants responded to standardized questionnaires and were examined by trained investigators. Smokers were defined as individuals who had smoked daily for at least 6 months during their life. Alcohol drinkers were defined as individuals who drank alcohol products at least once a week.

### Cardiovascular disease risk factors

In the morning before breakfast, participants’ body weight, height, blood pressure (BP), and waist circumference (WC) were measured. Body height and weight were accurate to 0.1 cm and 0.1 kg, respectively. Body mass index (BMI) was calculated by dividing body weight (kg) by the height (m) squared. WC was measured as the smallest circumference between the rib margin and iliac crest. In addition, a venous blood sample was drawn after a 12-hour fast. Local laboratories, approved by national or provincial quality control systems already in place, tested fasting blood glucose (FG). Blood samples were shipped in dry ice to the Chinese Center for Disease Control and Prevention, where plasma triglycerides (TG), and high-density lipoprotein (HDL) cholesterol were measured enzymatically with a Hitachi 7060, 7180 auto-analyzer. The participants’ blood pressures were measured with standardized mercury sphygmomanometers. Two consecutive readings of BP were taken on the right arm according to the 1999 World Health Organization/International Society of Hypertension guidelines on hypertension [30] with the participant in a seated position after 5 minutes of rest; the mean of the 2 readings was used for analysis.

The primary outcome was CVD risk factors that were defined by the following conditions: abdominal obesity (WC ≥ 85 cm) [31]; hypertension (systolic blood pressure ≥140 mmHg or/and diastolic blood pressure ≥ 90 mmHg) [32], [33]; hyperglycemia (FG≥ 6.1 mmol/L) [34]; elevated triglyceride (TG ≥ 5.18 mmol/L) [35]; and low HDL (HDL < 1.03 mmol/L) [35].

According to the International Diabetes Federation (IDF), the definition of metabolic syndrome was WC > 90 cm plus any two or more of the following risk factors: 1) TG > 1.7 mmol/l or specific treatment for this abnormality; 2) HDL cholesterol < 1.03 mmol/l in men or < 1.29 mmol/l in women or specific treatment for this abnormality; 3) BP >130/85 mmHg or treatment of previously diagnosed HT; and 4) FG >5.6 mmol/l or previously diagnosed diabetes [36].

### Statistical analyses

Initially, we analyzed the individual associations of PAL and dietary patterns with CVD risk factors by estimating odds ratios (OR) and 95% confidence intervals (CI) in the multivariable logistic models. We established 2 multivariate models. The first model was adjusted for age (single year), living area (urban area was defined as an urban district, city or town with a population density higher than 1,500/km^2^; the remaining area was defined as a rural area.), education level (uneducated, primary school, middle school, higher education), annual income per family member (<800, 800-1999, 2000-4999, ≥5000 RMB, RMB is the abbreviation of the Chinese currency unit (RenMinBi)), smoking status (yes/no), and alcohol consumption (yes/no). The second model was further adjusted for BMI because it is by far a very strong predictor of CVD risk factors. Next, ORs and 95% CIs evaluated joint categories of dietary patterns and physical activity levels in relation to CVD risk factors by logistic models adjusted for the covariates previously mentioned. Finally, we defined the very active PAL and the Green Water dietary pattern as the low-risk group and created a binary variable: the participants received a value of 1 if he or she met the criteria for low risk and a value of 0 otherwise. ORs and 95% CIs for the individual and joint associations in relation to CVD risk factors of the binary variables were estimated by logistic model adjusted for the covariates mentioned above. Population-attributable risk (PAR) and 95% CI were estimated based on the parameters from the multivariate logistic models using the implicit delta method developed by Benichou and Gail in 1990 [37]–[39], which has already been used in other well-known epidemiologic studies [40], [41]. All the analyses were conducted with SAS version 8.2 (SAS Institute, Cary, NC, USA). All *P* values were 2-tailed (α = 0.05).

## Results

Table 1 shows the selected characteristics of all participants according to the different dietary patterns and 4 PALs. For each dietary pattern, the distribution of adopters was 39.8% for the Green Water pattern, 27.1% for the Yellow Earth pattern, 14.4% for the Western Adopter pattern, and 18.8% for the New Affluent pattern. The Green Water pattern adopters were older and characterized by a lower education level, having a higher consumption of rice and vegetables and a moderate consumption of animal sourced foods. The Yellow Earth pattern adopters were characterized by living in rural areas with a lower education and economic level; they had a higher consumption of wheat flour, tubers, and other cereals, and a lower consumption of fresh vegetables, fruit, and animal sourced food, and were more likely to smoke. The New Affluent pattern adopters had a higher intake of animal sourced foods and soybean products, and were characterized by living in urban areas. The Western Adaptor pattern adopters were younger, had a higher education and economic level and were characterized by living in urban areas; they were more likely to consume alcohol, animal sourced food, cakes, and beverages. The percentage of the participants whose PAL was sedentary, low active, active and very active was 16.1%, 12.6%, 22.2% and 49.1%, respectively. The individuals whose PAL was very active and active were characterized by living in rural areas, having a lower education and economic level, and more likely to smoke. The participants who had sedentary and low active PAL were characterized by living in urban areas, having a higher education and economic level.

**Table 1:** Selected characteristic of 13511 Chinese male adults according to dietary patterns and physical activity levels (%).

		Dietary Pattern				Physical Activity Level			
	All	Green Water	Yellow Earth	Western Adopter	New Affluent	Very Active	Active	Low Active	Sedentary
n (%)	13511 (100.0)	5372 (39.8)	3655 (27.1)	1948 (14.4)	2536 (18.8)	6630 (49.1)	3004 (22.2)	1701 (12.6)	2176 (16.1)
Age (years)									
18–44	61.0	58.8	59.0	70.9	61.0	60.9	62.2	58.6	61.4
45–59	39.0	41.3	41.0	29.1	39.0	39.1	37.8	41.4	38.6
Living area									
Rural	74.8	81.7	91.0	47.9	57.4	84.8	81.6	57.1	48.2
Urban	25.2	18.3	9.0	52.1	42.6	15.2	18.4	42.9	51.8
Annual income (RMB)									
<800	13.7	12.8	23.7	3.9	8.5	16.9	16.3	7.5	5.0
800-1999	32.7	35.5	44.4	16.1	22.5	37.0	39.5	24.2	16.8
2000-4999	30.4	33.3	24.5	31.0	32.6	30.9	28.5	30.1	31.9
≥5000	23.2	18.4	7.4	49.0	36.4	15.2	15.7	38.2	46.3
Educational level									
Uneducated	29.3	38.8	32.1	14.9	16.8	35.9	31.9	19.2	13.4
Primary school	43.9	42.2	50.9	36.4	43.2	47.3	47.5	37.9	33.3
Middle school	18.7	14.9	14.5	28.3	25.5	14.4	15.5	23.3	33.1
Higher education	8.0	4.2	2.5	20.4	14.5	2.4	5.1	19.5	20.1
Smoking status									
No	39.5	39.5	38.2	39.7	41.1	37.4	39.8	42.2	43.4
Yes	60.5	60.5	61.8	60.3	58.9	62.6	60.2	57.8	56.6
Alcohol consumption									
No	53.3	50.8	62.6	45.0	51.3	52.4	55.8	52.2	53.3
Yes	46.7	49.2	37.4	55.0	48.7	47.6	44.2	47.8	46.7

The relationship between the dietary patterns and CVD risk factors was examined by estimating the prevalence and OR for adopting each dietary pattern (Table 2). The Green Water pattern adopters had the lowest prevalence for abdominal obesity (AO), hypertension (HT), hyperglycemia (HG), elevated triglyceride (ETG), low HDL (LHDL), and metabolic syndrome (MS).

**Table 2:** Prevalence and odds ratio (95% confidence interval) of CVD risk factors according to dietary pattern among 13511 Chinese male adults.

	Dietary pattern				*P* for trends
	Green Water	Yellow Earth	Western Adopter	New Affluent	
Abdominal obesity					
Prevalence	19.0	23.1	36.7	39.3	<.0001
Odds ratio (95% CI)*	1.0	1.60 (1.43–1.78) §	1.56 (1.37–1.77) §	2.01 (1.79–2.25) §	<.0001
Odds ratio (95% CI)†	1.0	1.24 (1.05–1.47) §	1.23 (1.02–1.49) §	1.00 (0.85–1.18)	0.005
Hypertension					
Prevalence	29.3	34.4	30.7	39.2	<.0001
Odds ratio (95% CI)*	1.0	1.40 (1.27–1.54) §	1.17 (1.03–1.33) §	1.61 (1.44–1.79) §	<.0001
Odds ratio (95% CI)†	1.0	1.18 (1.07–1.31) §	1.04 (0.91–1.18)	1.31 (1.17–1.47) §	<.0001
Hyperglycemia					
Prevalence	3.5	3.7	5.1	7.5	<.0001
Odds ratio (95% CI)*	1.0	1.20 (0.95–1.52)	1.21 (0.92–1.58)	1.71 (1.36–2.14) §	<.0001
Odds ratio (95% CI)†	1.0	1.01 (0.79–1.28)	1.05 (0.80–1.39)	1.38 (1.10–1.73) §	0.008
Elevated TG					
Prevalence	13.4	13.9	20.6	18.1	<.0001
Odds ratio (95% CI)*	1.0	1.15 (1.01–1.31) §	1.26 (1.09–1.47) §	1.17 (1.02–1.35) §	0.006
Odds ratio (95% CI)†	1.0	0.91 (0.79–1.04)	1.09 (0.93–1.27)	0.86 (0.75–1.00)	0.136
Low HDL					
Prevalence	15.8	26.3	23.3	25.0	<.0001
Odds ratio (95% CI)*	1.0	1.84 (1.64–2.05) §	1.49 (1.30–1.72) §	1.66 (1.46–1.87) §	<.0001
Odds ratio (95% CI)†	1.0	1.61 (1.44–1.80) §	1.36 (1.18–1.57) §	1.38 (1.22–1.57) §	<.0001
Metabolic syndrome					
Prevalence	6.8	9.4	14.6	16.6	<.0001
Odds ratio (95% CI)*	1.0	1.72 (1.46–2.03) §	1.69 (1.41–2.02) §	2.08 (1.77–2.44) §	<.0001
Odds ratio (95% CI)†	1.0	1.22 (1.01–1.48) §	1.38 (1.12–1.70) §	1.36 (1.13–1.64) §	0.001

* Multivariable model adjusted for age (single year), living area (urban/rural), education level (uneducated/primary school/middle school/higher education), annual income per family member (<800/800-1999/2000-4999/≥5000 RMB), smoking status (yes/no), and alcohol consumption (yes/no).

† Further adjusted BMI (continuous).

§ *P*<0.05.

The participants with the New Affluent pattern had the highest prevalence for AO (39.3%), HT (39.2%), HG (7.5%), and MS (16.6%), whereas the participants adopting the Western Adopter and the Yellow Earth patterns held the highest prevalence of ETG (20.6%) and LHDL (26.3%), respectively. After simultaneous adjustment for age, living area, education level, annual income per family member, smoking status, and alcohol consumption, the New Affluent pattern adaptors demonstrated the highest likelihood of 5 CVD risk factors. Compared to the Green Water pattern, the OR for prevalence of AO, HT, HG, ETG and MS for the New Affluent pattern was 2.01 (95% CI, 1.79–2.25), 1.61 (95% CI, 1.44–1.79), 1.71 (95% CI, 1.36–2.14), 1.17 (95% CI, 1.02–1.35) and 2.08 (95% CI, 1.77–2.44), respectively. The Green Water pattern adopters had 50.2%, 37.9%, 41.5%, 14.5%, 39.8%, and 51.9% lower likelihood for AO, HT, HG, ETG, LHDL, and MS, respectively, compared to the New Affluent pattern adopters. After further adjusting for BMI in the multivariate model, the associations of the dietary patterns with the prevalence of AO, HT, HG, LHDL, and MS were attenuated, but not materially changed (Table 2). However, the significant associations between dietary patterns and prevalence of ETG disappeared after further adjustment for BMI (*P* for trend  =  0.1360).

Table 3 shows the associations between PAL and prevalence of CVD risk factors. The higher PAL was associated with lower prevalence of each CVD risk factor (*P* for trend <.0001). After simultaneous adjustment for age, living area, education level, annual income per family member, smoking status, and alcohol consumption, individuals who had the very active PAL presented 37.6%, 15.1%, 29.4%, 24.2%, 26.5%, and 42.1% lower likelihood for AO, HT, HG, ETG, LHDL, and MS, respectively, compared with the sedentary group. After further adjusting for BMI, the inverse associations of PAL with prevalence of AO, HG, ETG, LHDL, and MS were only appreciably attenuated, but remained significant. However, there was no longer a significant association between PAL and prevalence of HT after further adjustment for BMI (*P* for trend  =  0.322).

**Table 3:** Prevalence and odds ratio (95% confidence interval) of CVD Risk Factors According to Physical Activity Level among 13 511 Chinese Male Adults.

	Physical activity level				*P* for trends
	Very active	Active	Low active	Sedentary	
Abdominal obesity					
Prevalence	20.3	24.0	35.7	41.4	<.0001
Odds ratio (95% CI)*	1.0	1.20 (1.08–1.34) §	1.41 (1.24–1.61) §	1.60 (1.42–1.81) §	<.0001
Odds ratio (95% CI)†	1.0	1.20 (1.03–1.41) §	1.48 (1.22–1.78) §	1.54 (1.30–1.83) §	<.0001
Hypertension					
Prevalence	31.4	33.2	33.5	35.6	<.0001
Odds ratio (95% CI)*	1.0	1.12 (1.01–1.23) §	1.06 (0.94–1.20)	1.18 (1.05–1.32) §	0.007
Odds ratio (95% CI)†	1.0	1.08 (0.99–1.19)	1.00 (0.88–1.14)	1.07 (0.95–1.21)	0.322
Hyperglycemia					
Prevalence	3.5	4.0	6.0	7.1	<.0001
Odds ratio (95% CI)*	1.0	1.04 (0.82–1.31)	1.22 (0.94–1.58)	1.42 (1.12–1.80) §	0.004
Odds ratio (95% CI)†	1.0	1.00 (0.79–1.27)	1.16 (0.89–1.51)	1.28 (1.01–1.63) §	0.038
Elevated TG					
Prevalence	13.5	13.8	18.1	21.7	<.0001
Odds ratio (95% CI)*	1.0	0.99 (0.87–1.12)	1.11 (0.95–1.30)	1.32 (1.15–1.52) §	0.0002
Odds ratio (95% CI)†	1.0	0.94 (0.82–1.08)	1.05 (0.89–1.23)	1.18 (1.02–1.37) §	0.039
Low HDL					
Prevalence	19.2	21.2	25.2	25.6	<.0001
Odds ratio (95% CI)*	1.0	1.09 (0.98–1.22)	1.36 (1.19–1.56) §	1.36 (1.20–1.55) §	<.0001
Odds ratio (95% CI)†	1.0	1.06 (0.95–1.19)	1.32 (1.15–1.52) §	1.26 (1.11–1.44) §	<.0001
Metabolic syndrome					
Prevalence	7.6	9.2	13.9	18.2	<.0001
Odds ratio (95% CI)*	1.0	1.19 (1.02–1.40) §	1.34 (1.12–1.60) §	1.73 (1.47–2.03) §	<.0001
Odds ratio (95% CI)†	1.0	1.15 (0.96–1.39)	1.24 (1.01–1.53) §	1.57 (1.31–1.90) §	<.0001

* Multivariable model adjusted for age (single year), living area (urban/rural), education level (uneducated/primary school/middle school/higher education), annual income per family member (<800/800-1999/2000-4999/≥5000 RMB), smoking status (yes/no), and alcohol consumption (yes/no).

† Further adjusted BMI (continuous).

§ *P*<0.05.

**Figure 1:**
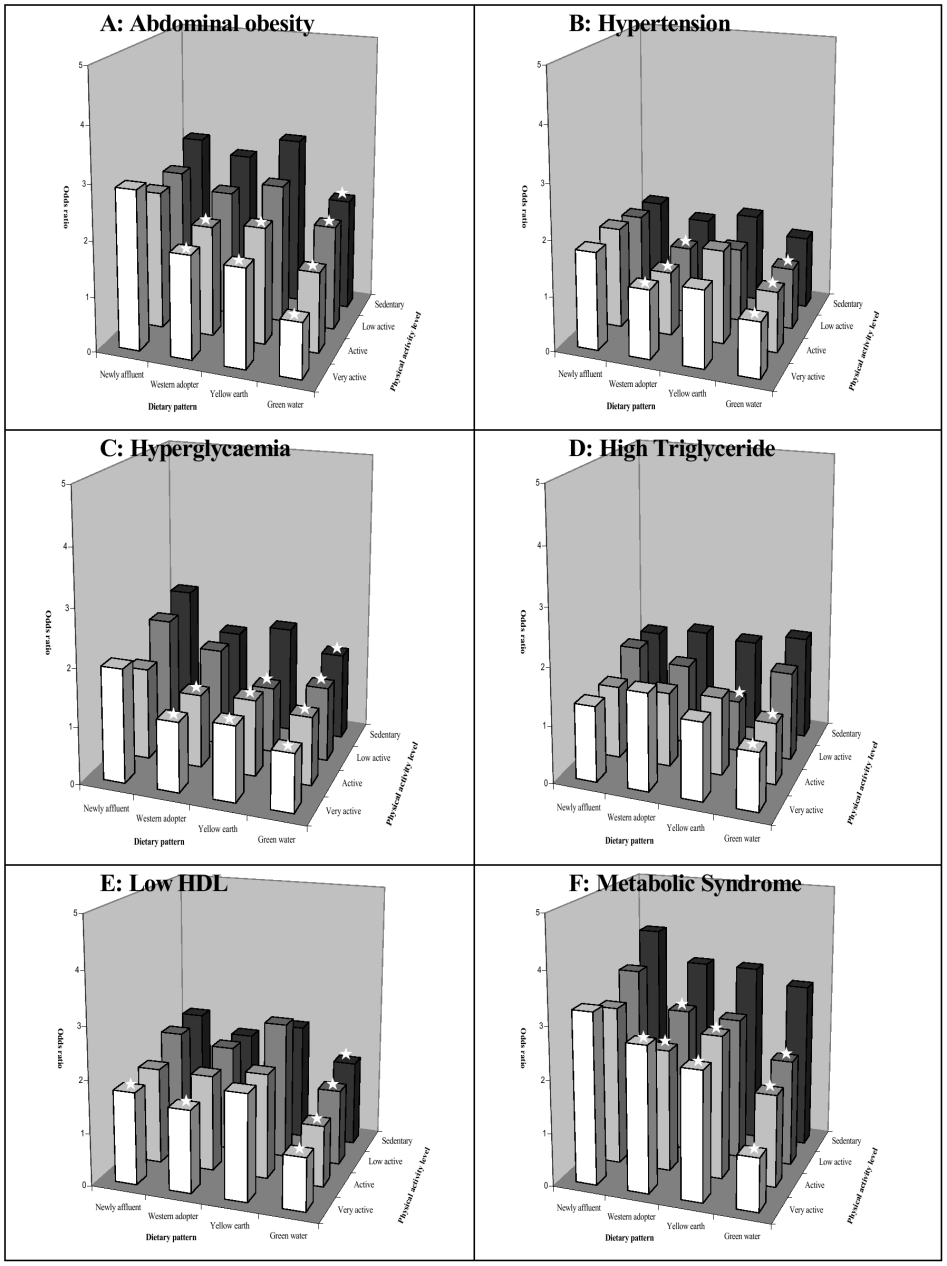
Joint association of dietary pattern and physical activity with the likelihood of CVD risk factors.

The joint associations of PAL and dietary pattern with prevalence of CVD risk factors are shown in Figure 1. The participants who adopted both the very active PAL and the Green Water dietary pattern had the lowest OR for prevalence of AO (*P* for trend <.0001), HT (*P* for trend  =  0.0004), HG (*P* for trend  =  0.0009), ETG (*P* for trend <.0001), LHDL (*P* for trend <.0001), and MS (*P* for trend <.0001). The OR for prevalence of AO, HT, HG, ETG, LHDL, and MS was 0.40 (95% CI 0.34–0.48), 0.64 (95% CI 0.54–0.76), 0.42 (95% CI 0.30–0.58), 0.64 (95% CI 0.52–0.79), 0.61 (95% CI 0.52–0.72), and 0.32 (95% CI 0.25–0.40), respectively, compared with individuals who adopted both the sedentary PAL and the New Affluent dietary pattern.

Table 4 shows PARs for the participants in the low-risk groups for dietary pattern and PAL both individually and in combination. Different proportions of cases for AO (31%), HT (17%), HG (18%), ETG (11%), LHDL (29%), and MS (34%) might have been potentially prevented if all the participants followed the Green Water dietary pattern. The cases of AO, HT, HG, ETG, LHDL, and MS at 20%, 17%, 5%, 10%, 8%, 11%, and 20%, respectively, could be attributable to not being very physically active. Adopting both the very active PAL and the Green Water dietary pattern might potentially prevent different proportions of cases for AO (54%), HT (27%), HG (31%), ETG (28%), LHDL (45%), and MS (63%) when analyzing this sample in combination.

**Table 4:** Odds ratio (95% confidence interval) and population attributable risk (95% confidence interval) of CVD risk factors according to dietary pattern and physical activity level among 13511 Chinese male adults.

	No. of Cases	Odds Ratio* † (95% Confidence Interval)	Population Attributable Risk (95% Confidence Interval) * †, %
Abdominal obesity			
Green Water dietary pattern	1345	0.58 (0.54–0.64)	31 (27–35)
Very active physical activity	1022	0.73 (0.67–0.80)	20 (16–24)
Green Water dietary pattern + Very active physical activity	419	0.47 (0.42–0.53)	54 (50–59)
Hypertension			
Green Water dietary pattern	2082	0.71 (0.66–0.77)	17 (13–21)
Very active physical activity	1574	0.89 (0.83– 0.97)	5 (1–9)
Green Water dietary pattern + Very active physical activity	852	0.71 (0.64–0.78)	27 (21–32)
Hyperglycemia			
Green Water dietary pattern	235	0.73 (0.61–0.88)	18 (7–28)
Very active physical activity	188	0.84 (0.70–1.01)	10 (1–20)
Green Water dietary pattern + Very active physical activity	87	0.68 (0.53–0.87)	31 (16–45)
Elevated TG			
Green Water dietary pattern	898	0.84 (0.76–0.94)	11 (5–17)
Very active physical activity	722	0.90 (0.81–0.99)	8 (3–13)
Green Water dietary pattern + Very active physical activity	339	0.73 (0.64–0.83)	28 (20–36)
Low HDL			
Green Water dietary pattern	1275	0.59 (0.53–0.64)	29 (25–34)
Very active physical activity	849	0.82 (0.75–0.90)	11 (7–15)
Green Water dietary pattern + Very active physical activity	414	0.55 (0.49–0.62)	45 (39–51)
Metabolic syndrome			
Green Water dietary pattern	503	0.55 (0.48–0.62)	34 (28–40)
Very active physical activity	364	0.72 (0.64–0.82)	20 (14–26)
Green Water dietary pattern + Very active physical activity	127	0.37 (0.31–0.45)	63 (53–70)

* Compared with all other participants not in this low-risk group.

† Multivariable model adjusted for age (single year), living area (urban/rural), education level (uneducated/primary school/middle school/higher education), annual income per family member (<800/800-1999/2000-4999/≥5000 RMB), smoking status (yes/no), alcohol consumption (yes/no).

## Discussion

In this nationally representative sample of Chinese males, we observed that the Green Water dietary pattern, characterized by high intake of rice, vegetables, and moderate intake of animal sourced foods, was associated with the lowest prevalence of CVD risk factors among all four patterns. Compared with the Green Water dietary pattern, the New Affluent dietary pattern, characterized by a higher intake of animal sourced foods and soybean products, was associated with increased prevalence of CVD risk factors. We also found an inverse and graded association between PAL and prevalence of CVD risk factors. In addition, when analyzed in combination, the very active PAL and the Green Water dietary pattern presented a remarkable potential for CVD risk factor prevention.

The four major dietary patterns reflect the ongoing nutrition transition which has accompanied the rapid economic development, urbanization and subsequent lifestyle transition in China since the early 1980s [42]. The Green Water pattern represents a traditional Chinese dietary pattern in southern China, where rice is the major staple food. Older generations and rural residents are more likely to adopt this pattern. Previous studies [43], [44] have reported that the prevalence of CVD risk factors, such as overall obesity, abdominal obesity, dyslipidemia, hypertension and type 2 diabetes, was significantly lower in southern and rural areas of China and suggested that the geographic variation in CVD risk factors was mainly explained by the dietary factors. Our findings are consistent with the previous studies. The low prevalence of CVD risk factors associated with the Green Water dietary pattern may be explained by the high consumption of protective food items, such as vegetables, fruits, aquatic products, soybean products and nuts. The cluster of protective food items in connection with other healthy dietary patterns has proved to be effective when preventing CVD and its risk factors in western [8]–[13] and eastern Asian populations [40], [45], [46]. Similar dietary patterns have been reported in our previous studies and a study in southern China [47] and [48], [49]. The potential of Green Water pattern for CVD risk factor prevention is further supported by a Japanese traditional dietary pattern study [46]. In this study, individuals who adopted a Japanese traditional dietary pattern, which was characterized by higher intake of soybean products, fish, seaweeds, vegetables, fruits, and moderate intake of pork and poultry, had lower levels of systolic blood pressure (SBP), diastolic blood pressure (DBP), TG, and LDL, as well as higher level of HDL, compared to the Western dietary pattern adopters. The Yellow Earth pattern is another traditional dietary pattern typically adopted by residents living in northern China, where wheat is the major staple food. Similar to the Green Water pattern, the Yellow Earth pattern is also more likely to be followed by older individuals who were living in rural areas and have a lower socioeconomic level. Even though the Yellow Earth pattern adopters consume very limited animal sourced food, they present significantly higher prevalence of CVD risk factors, compared with the Green pattern adopters. To date, substantial evidence is still lacking for an explanation of this pattern’s detrimental effect on CVD risk factors. We assume that the high intake of refined carbohydrates and low consumption of fresh vegetables, fruits and aquatic products may contribute to the development of CVD risk factors [12], [50]. Our assumption can be partly supported by a traditional Korean dietary pattern study. Kim et al. [45] found that adaptors of the traditional Korean dietary pattern, in which cereal products and starchy tubers were also the major staple portion, did not show significantly lower prevalence of HT when compared with the western dietary pattern adopters. Kim suggested that the dietary practice of Koreans who consumed salted vegetables instead of fresh vegetables might explain this phenomenon. We observed Western-style changes in dietary habits of the New Affluent and Western Adopter pattern adopters; for instance, they tend to consume more meat and sugar-sweetened beverages instead of the traditional cereal based, low-fat and high vegetable content stir-fried meals. The adopters of these two patterns were younger, had higher socioeconomic level and lived in urban areas. The higher prevalence of CVD risk factors relating to the two dietary patterns is consistent with the relationship between CVD risk factors and western dietary patterns observed by previous studies in the western populations 8–13. However, it is noteworthy that the associations between the two westernized dietary patterns and prevalence of CVD risk factors are not as strong as that observed in the western populations [8]–[13]. These associations may be explained the relatively high consumption of plant sourced foods, such as fruit, vegetable and soybean products in the New Affluent and Western Adopter patterns; these two westernized dietary patterns are somewhat different from the western dietary pattern (high in red meat, processed meat, refined grains, sweets and deserts, French fries, and high-fat dairy products) summarized in the western populations.

According to a majority of epidemiological studies, active physical activity level has been convincingly associated with decreased risk of CVD [6], [16], [51] and is often included as a standard clinical recommendation for patients with CVD risk factors [16], [51]. In the present study, we employed PAL, which provides overall information about frequency, duration and intensity of the five domains of physical activity, and observed independent graded inverse associations between PAL and CVD risk factors. The magnitude of our findings is further supported by other studies [52]–[63]. As for the relation between PAL and abdominal obesity, the Inter99 Study [52] in Denmark found that a five-year change in PAL was significantly and independently associated with change in waist circumference. In those who increased their PAL from baseline to five-year follow-up, a 4.2 cm decrease in waist circumference was observed. Previous studies on physical activity and serum lipids presented inconsistent results. Our findings of inverse associations between PAL and prevalence of dyslipidemia were consistent with the majority of previous findings [52]–[57]. Kronenberg et al. [53] reported that leisure time physical activity was positively associated with HDL cholesterol among a population-based sample of 1778 subjects from the NHLBI Family Heart Study. In the extreme physically vigorous group, the EPIC-Norfolk cohort study [54] observed that the mean value of TG was 0.22 mmol/L higher, and the mean value of HDL was 0.08 mmol/L lower than in the most sedentary group of male subjects. As for the relationship between PAL and HT, our study failed to detect a significant association, as did several other studies [53], [58], [59], [60]. For example, in a population-based sample from the 3-year follow-up of the Inter 99 study [60] in Denmark, no association was found between physical activity and SBP or DBP. We assume that confounding caused by other factors not measured and therefore not adjusted for (eg. stress) may explain the lack of association between PAL and HT. Several guidelines for diabetes prevention [51], [61] have highlighted physical activity as an essential part of lifestyle intervention for reducing risk of diabetes. Our findings of inverse association between PAL and hyperglycemia correspond with considerable epidemiological evidence which has validated these guidelines. In a multi-ethnic population, Dowse et al. [62] found that higher PAL was associated with lower prevalence of both impaired glucose tolerance (IGT) and type 2 diabetes. For the low PAL group, the multivariable-adjusted odds ratio estimates of IGT and type 2 diabetes were 1.31 and 1.70, respectively, compared with the heavy physical activity group. In relation to MS, our findings of an inverse association between PAL and MS are consistent with other studies [63]–[65]. In a study based on the 2003–04 and 2005–06 cycles of the U.S. National Health and Nutrition Examination Survey (NHANES), the physically inactive group showed a decreased odds ratio of MS as compared to the physically very active group [63].

Our data point to a potential joint effect between the healthy dietary pattern and vigorous physical activity as an integral strategy for CVD risk factor prevention. The combined effect of dietary and physical activity intervention on CVD risk has been studied in the Diabetes Prevention Program (DPP) trial that included overweight and obese adults with both elevated fasting glucose levels and elevated 2-hour post-challenge glucose levels. Structured advice for adopting a healthy low-calorie diet and being moderately active improved multiple cardio-metabolic risk factors, including blood pressure, triglycerides, HDL cholesterol, reduced incidence of HT, diabetes, and dyslipidemia [66]–[68]. Notably, our findings are consistent with the results observed by DPP and other randomized trials [69], suggesting that the potentially significant benefits of diet and physical activity for preventing CVD risk factors in a high-risk population extend to a much broader male population. Furthermore, several observational studies [20]–[23], [70] also support our findings. For example, in the Cardiovascular Health Study, Mozaffarian et al. [70] found that active PAL (26%) and healthy dietary habits (31%) were independently associated with significantly lower risk for diabetes among older adults. In combination, these two, low-risk lifestyle factors were associated with a 46% lower risk of diabetes. Additionally, 40% of cases of diabetes appeared to be attributable to the combination of these two lifestyle risk factors.

The strengths of our analysis include the extensive information on lifestyle factors, the high quality of the data collected, and the national representative sample size available for analysis, providing us a population-based sample of Chinese men and increasing generalizability. In addition, both dietary pattern and PAL were measured by validated methods which were tested among the Chinese population in the previous studies [14], [19], [26], [28], [29].

Potential limitations should be considered. By using a cross-sectional study, we cannot formally draw a conclusion about causality. However, of note, when lifestyle factors, such as diet and physical activity, and CVD risk factors are investigated, the reverse causality is unlikely to play a role. Data on dietary intake and physical activity were collected by questionnaires that recalled information over 1 year. Moreover, dietary and physical activity habits may change over a lifetime, and these changes may have an additional impact on CVD risk factors. Although we adjusted for major socio-demographic characteristics and lifestyle factors simultaneously, residual confounding by unknown or unmeasured factors may be present. However, given the magnitude of the estimated risk and consistency of our results with previous studies, it is improbable that all of the observed risk differences are owing to residual confounding. It is possible that the diagnosis of CVD risk factors could be related to lifestyle. Individuals with healthier lifestyles might be more willing to participate in our survey’s physical examinations, causing overestimation of CVD risk factor prevalence among those with healthier lifestyles. Dichotomization of some socio-demographic and lifestyle-controlled variables (age, region, smoking status and alcohol consumption) that have graded effects on risk could also have attenuated the magnitude of association compared with other comparisons. Therefore, our findings likely underestimate the importance of each of the individual lifestyle factors and their combined effects on CVD risk factors.

In conclusion, adherence to a combination of low-risk dietary and physical activity habits was associated with a remarkably lower prevalence of CVD risk factors, and might have the potential to prevent a large proportion of cases of CVD risk factors among Chinese males. Unfortunately, following the rapid economy development and urbanization, Chinese lifestyle has changed, becoming especially westernized [42], [71]. Our study found that nearly 1 out of 5 participants adopted the least CVD healthy dietary pattern. Moreover, a previous study reported that 31.7% of Chinese adults were physically insufficient [32]. The prevalence of adverse lifestyle risk factors is worrying, but at the same time may provide a tremendous potential for improving the treatment and prevention strategies in China. Therefore, the new guidelines in CVD intervention and prevention should emphasize healthy and achievable dietary and physical activity goals equally for both the individual and the population.

## Supporting Information

Table S1 **Food consumption of 13 511 Chinese male adults according to dietary patterns.**
(DOC)

Table S2 **Nutrition intake of 13 511 Chinese male adults according to dietary patterns.**
(DOC)

Text S1 **Explanations of physical activity level.**
(DOC)
