# Supplementary material for: Joint Association of Dietary Pattern and Physical Activity Level with Cardiovascular Disease Risk Factors among Chinese Men: A Cross-Sectional Study
Source: PLoS One. 2013 Jun 19;8(6):e66210. doi: 10.1371/journal.pone.0066210 (PMC3686814; doi:10.1371/journal.pone.0066210)
Supplement: Table S1 — Food consumption of 13 511 Chinese male adults according to dietary patterns. (DOC) [file pone.0066210.s001.doc]

**Table S1. Food consumption of 13 511 Chinese male adults according to dietary patterns (Mean ± Standard Deviation, g/day)**

|  | Dietary Pattern | | | |
| --- | --- | --- | --- | --- |
|  | Green Water | Yellow Earth | Western Adopter | New Affluent |
| Rice and rice products | 463.8±173.3 | 101.3±137.9 | 279.5±172.1 | 165.8±144.5 |
| Wheat and wheat products | 30.8±65.2 | 314.8±208.2 | 119.8±153.5 | 250.0±211.8 |
| Other cereals | 5.5±35.3 | 66.5±95.8 | 15.7±32.6 | 34.4±61.9 |
| Starchy tubers | 23.7±55.6 | 82.1±114.7 | 33.2±51.1 | 55.7±83.0 |
| Fried wheat products | 3.3±12.4 | 12.3±29.0 | 14.8±30.1 | 19.0±31.1 |
| Pork | 51.4±60.5 | 16.4±32.4 | 69.9±67.0 | 51.5±65.0 |
| Beef/lamb | 3.3±12.3 | 2.9±16.2 | 15.5±33.1 | 17.4±36.3 |
| Poultry | 8.7±20.3 | 1.6±6.2 | 17.1±21.1 | 13.5±28.0 |
| Aquatic product | 23.1±42.2 | 3.2±19.8 | 34.4±44.7 | 25.8±44.6 |
| Milk | 3.2±24.7 | 5.5±39.6 | 47.9±96.8 | 62.1±130.8 |
| Egg | 21.5±27.1 | 33.3±37.1 | 40.2±36.3 | 51.1±42.7 |
| Soybean products | 41.7±59.5 | 30.8±43.4 | 69.1±72.7 | 68.6±83.3 |
| Dry bean | 4.9±21.3 | 5.0±22.6 | 9.3±24.9 | 6.8±25.8 |
| Fresh vegetables | 335.0±200.7 | 196.3±165.3 | 268.5±168.3 | 246.4±170.1 |
| Dry vegetables | 3.6±19.0 | 3.3±19.8 | 4.4±20.8 | 4.7±24.9 |
| Cake | 3.2±13.2 | 2.1±8.8 | 17.9±30.1 | 10.6±24.6 |
| Fruit | 44.5±72.3 | 52.6±71.6 | 105.5±99.3 | 107.9±103.5 |
| Nuts | 3.5±14.8 | 2.4±8.9 | 10.7±21.9 | 9.0±22.8 |
| Juice | 3.8±25.7 | 1.7±15.5 | 38.2±71.1 | 5.6±34.8 |
| Other beverages | 9.4±53.9 | 3.5±34.5 | 42.7±88.2 | 24.9±102.1 |
| Vegetable oil | 23.8±21.4 | 23.8±24.0 | 32.2±22.0 | 34.7±23.3 |
| Animal fat | 14.3±18.1 | 6.7±12.7 | 5.4±11.9 | 5.5±11.9 |
