# Supplementary material for: Joint Association of Dietary Pattern and Physical Activity Level with Cardiovascular Disease Risk Factors among Chinese Men: A Cross-Sectional Study
Source: PLoS One. 2013 Jun 19;8(6):e66210. doi: 10.1371/journal.pone.0066210 (PMC3686814; doi:10.1371/journal.pone.0066210)
Supplement: Table S2 — Nutrition intake of 13 511 Chinese male adults according to dietary patterns. (DOC) [file pone.0066210.s002.doc]

**Table S2 Nutrition intake of 13 511 Chinese male adults according to dietary patterns (Mean ± Standard Deviation)**

|  | Dietary Pattern | | | |
| --- | --- | --- | --- | --- |
|  | Green Water | Yellow Earth | Western Adopter | New Affluent |
| Energy (kcal/day) | 2699.3±736.7 | 2438.7±815.9 | 2790.2±783.2 | 2844.4±825.0 |
| Carbohydrate (g/day) | 421.5±134.7 | 395.9±157.1 | 382.7±126.2 | 398.2±148.1 |
| Fat (g/day) | 68.5±32.0 | 56.8±29.1 | 88.2±35.6 | 87.3±35.0 |
| Protein (g/day) | 69.5±22.9 | 69.8±26.3 | 84.8±28.7 | 88.9±30.5 |
| Dietary fiber (g/day) | 12.2±5.4 | 16.4±7.4 | 14.2±6.4 | 16.6±7.4 |
| Sodium (mg/day) | 6002.7±3015.2 | 6319.2±3377.0 | 5989.1±3180.8 | 6514.9±3045.5 |
| Calcium (mg/day) | 540.8±253.4 | 488.8±248.1 | 659.9±316.7 | 704.9±326.1 |
| Iron (mg/day) | 25.0±8.2 | 27.3±10.3 | 28.0±9.6 | 30.5±10.6 |
| Zinc (mg/day) | 13.8±4.0 | 11.8±4.2 | 14.9±4.7 | 14.9±4.8 |
| Magnesium (mg/day) | 343.6±114.3 | 397.4±162.6 | 390.6±134.0 | 429.7±155.3 |
| Vitamin A (μg/day) | 123.4±147.2 | 106.7±110.0 | 269.7±226.2 | 267.9±208.8 |
| Thiamin (mg/day) | 1.0±0.3 | 1.5±0.6 | 1.3±0.5 | 1.5±0.6 |
| Riboflavin (mg/day) | 0.8±0.3 | 0.8±0.3 | 1.0±0.4 | 1.1±0.4 |
| Niacin (mg/day) | 16.2±4.9 | 13.7±5.2 | 17.2±5.6 | 17.0±5.9 |
| Vitamin C (mg/day) | 142.8±81.3 | 106.4±71.0 | 128.9±71.1 | 127.4±76.0 |
| Vitamin E (mg/day) | 24.7±15.2 | 29.1±16.0 | 34.0±17.3 | 37.9±17.8 |
