# Supplementary material for: Joint Association of Dietary Pattern and Physical Activity Level with Cardiovascular Disease Risk Factors among Chinese Men: A Cross-Sectional Study
Source: PLoS One. 2013 Jun 19;8(6):e66210. doi: 10.1371/journal.pone.0066210 (PMC3686814; doi:10.1371/journal.pone.0066210)
Supplement: Text S1 — Explanations of physical activity level. (DOC) [file pone.0066210.s003.doc]

Text S1 Explanations of physical activity level (PAL)

A sedentary PAL is set to reflect the basal energy expenditure (BEE), the thermal effect of food, and the physical activities that are required for an individual’s independent living. A low-active PAL for an adult weighing 70 kg is set to include an exertion equivalent to walking 2.2 mi/d at a rate of 3 to 4 mph or the equivalent energy expenditure in other activities, in addition to the activities that are part of independent living. The active PAL category was set at a PAL of 1.6 to 1.89. Mid-weight individuals with a PAL of 1.75 (midpoint in the active PAL category) would on average to be equivalent to walking 7 mi/d at the rate of 3 to 4 mph, while walking ~17 mi/d would be equivalent to the sum of the activities above independent living carried out by amid- weight individual with a PAL of 2.2 (midpoint in the very active PAL category).
